# The effect of vertical skeletal proportions, skeletal maturation, and age on midpalatal suture maturation: a CBCT-based study

**DOI:** 10.1186/s40510-023-00504-0

**Published:** 2024-02-05

**Authors:** Martina Ferrillo, Kieran Daly, Nikolaos Pandis, Padhraig S. Fleming

**Affiliations:** 1 Dentistry Unit, Department of Health Sciences, University of Catanzaro “Magna Graecia”, Catanzaro, Italy; 2 Dublin Dental University Hospital, Trinity College Dublin, The University of Dublin, Dublin, Ireland; 3 Northern Cross Dental, Dublin, Ireland; 4 Department of Orthodontics and Dentofacial Orthopedics, Dental School, Medical Faculty, University of Bern, Bern, Switzerland

**Keywords:** Rapid maxillary expansion, Cone-beam computed tomography, Midpalatal suture

## Abstract

**Background:**

Midpalatal suture (MPS) maturation may be influenced by a range of parameters including age, gender, and vertical skeletal pattern. We therefore aimed to evaluate the effect of Frankfurt-mandibular angle (FMA), skeletal maturation, and age on the timing of MPS maturation.

**Methods:**

In this cross-sectional study, cone-beam computed tomography (CBCT) and lateral cephalograms were used to assess the MPS and cervical vertebral maturation (CVM) stage. A proportional odds logistic regression model was used to assess associations between age adjusted for gender and MPS maturation, and a regression analysis was performed to analyze the effect of vertical pattern on these associations.

**Results:**

A total of 201 patients (84 male and 117 female) with a mean age of 13.48 (SD 1.94) were included. With increasing age, the odds of belonging to a higher maturation stage increased (OR: 2.14; 95% CI 1.789; 2.567; *P* < 0.001); however, no association between FMA and MPS maturation was observed (OR: 1.01; 95% CI 0.964; 1.051; *P* = 0.76). A strong correlation between MPS maturation and CVM stage was not reported. Males had a higher probability of belonging to a lower MPS maturation stage (OR: 0.24; 95% CI 0.136; 0.415; *P* < 0.001).

**Conclusions:**

Based on this cross-sectional analysis, midpalatal sutural maturation classification is associated with chronological age and occurs later in males. Neither CVM staging nor variation in vertical skeletal proportions were useful predictors of midpalatal maturation stage.

## Introduction

The treatment of transverse maxillary deficiency typically involves rapid maxillary expansion (RME) in order to open the midpalatal suture (MPS) by disrupting the sutural connective tissue and separating the hemi-maxillae [1]. In growing patients, the midpalatal suture is typically patent and therefore conducive to predictable separation [2]. As such, RME can be performed with tooth-borne appliances, through the application of heavy and intermittent forces over a brief period [3]. Earlier treatment has been recommended in order to facilitate occlusal correction, bypassing the effects of associated occlusal displacements, while also potentially influencing nasopharyngeal airway capacity at least in the short term [4, 5].

During growth, complex interdigitation appears in the midpalatal and circum-maxillary sutures increasing the mechanical resistance to effective RME [2]. Failure of separation may be accompanied by side effects, including pain, buccal tipping of the anchor teeth, gingival recession, and fenestration of the buccal cortical plate [6, 7]. In order to offset these undesirable side effects, micro-implant-assisted rapid palatal expansion (MARPE) has become popular, with reported success rates of approx. 85% in young adults [8, 9].

Given the variability in the timing of MPS fusion [10, 11], Angelieri et al. proposed a method for assessing sutural maturation based on cone-beam computed tomography (CBCT) by defining five maturational stages (A–E) [12, 13]. This classification was intended to reduce the risk of RME failure in older patients but also to curtail the use of surgically assisted rapid palatal expansion (SARPE) to skeletally mature patients (in stages D and E) [12–14].

Since Angelieri et al. reported limited correlation between chronological age and the degree of fusion, especially in young adults, skeletal age has instead been used as an indicator of the timing and rate of growth [15, 16]. Based on cervical vertebrae maturation (CVM) staging, stages D and E are not normally observed before CVM stage 4 [17]. However, there is inter-individual variation with MPS patency possible at later ages in males [14, 18]. This may relate to the earlier skeletal maturation that occurs in females, especially in the circum-pubertal period [19].

Hyperdivergent subjects may be associated with increased facial height, higher palatal vault, narrower maxilla, and a higher preponderance of pediatric sleep-disordered breathing [20]. The pubertal growth spurt has also been shown to be more prolonged in these subjects [21, 22]. Oliveira et al. hypothesized that the fusion of the MPS may be delayed in hyperdivergent patients illustrating a higher preponderance of stages B and C in adult dolichofacial subjects, although the sample was confined to adults over 18 years [23]. The aim of this study was therefore to evaluate possible associations between midpalatal suture maturation and overall skeletal maturation, vertical skeletal proportions, and chronological age.

## Material and methods

### Participants

In this retrospective cross-sectional study, CBCT scans and cephalograms were selected from patients referred to Northern Cross Dental, Dublin from January 2019 to December 2022. The inclusion criteria were: (a) high-quality CBCT and lateral cephalograms taken within a 3-month period; (b) late mixed or permanent dentition; (c) good general health; and (d) aged 8–20 years. Subjects with craniofacial malformations (including cleft lip or palate), lesions in the region of the suture, history of trauma or surgical procedures, previous or concomitant orthodontic treatment, and systemic diseases that may influence the growth or bone metabolism were excluded from the study. The present study was conducted according to the STrengthening the Reporting of OBservational studies in Epidemiology (STROBE) Guidelines [24]. The study protocol was approved by the Ethics Committee of the Dublin Dental University Hospital (DSREC2023/03).

### Outcomes

**Fig. 1:**
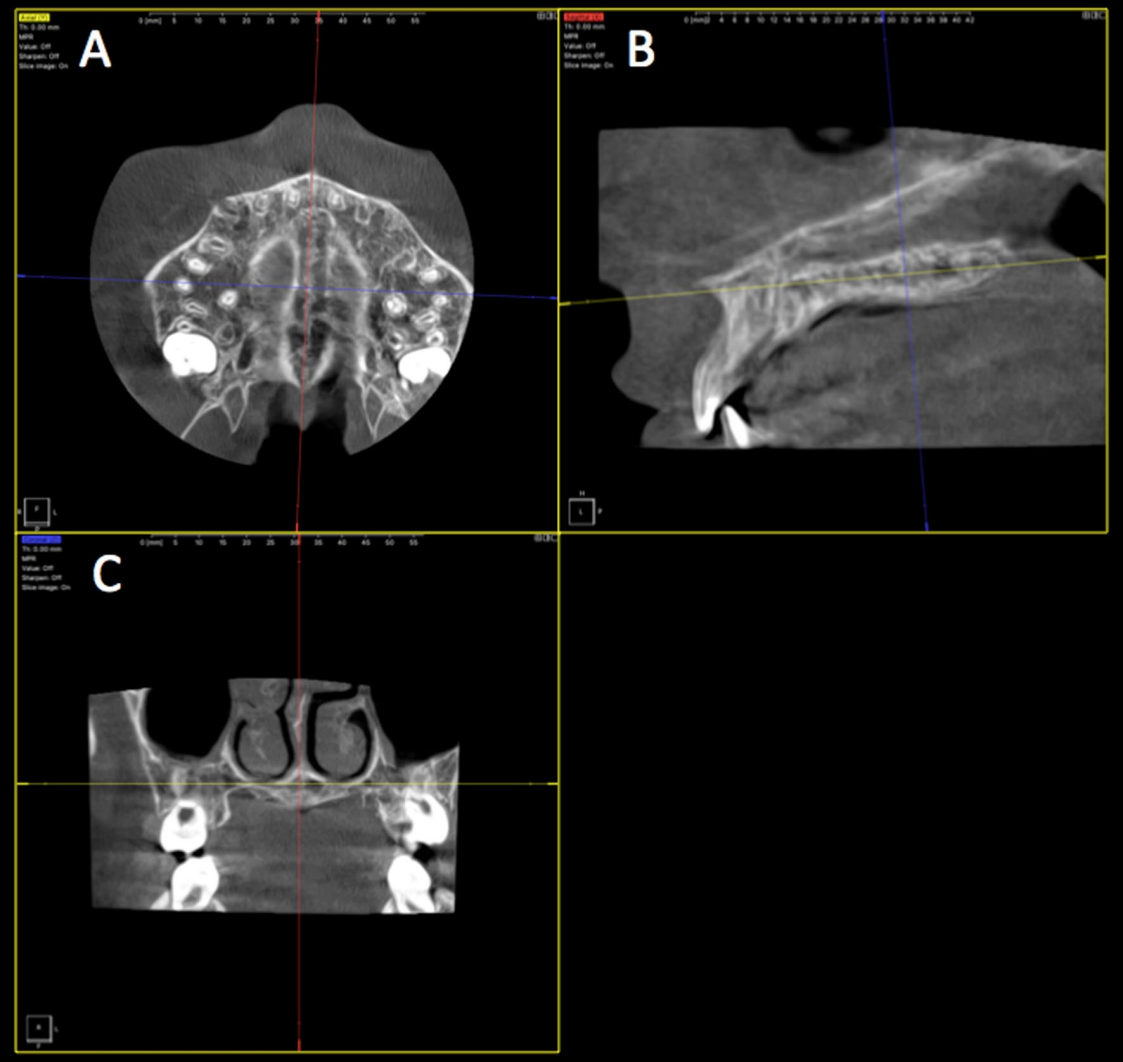
Assessment of the midpalatal suture (MPS) maturation in the axial (**A**), sagittal (**B**), and coronal (**C**) planes

Demographic data were collected for all participants. The evaluation of CBCT images was performed according to the qualitative methodology for individual evaluation of midpalatal suture maturation proposed by Angelieri et al. [12]. To achieve standardization of the head position in all three planes, the midsagittal cross-sectional slice was orientated parallel to the software horizontal axis, in order to position the palate horizontally. The central cross-sectional slice passing through the center of the hard palate was selected for sutural maturation assessment (Fig. 1). Two axial cross-sectional slices were selected in the presence of a thick or curved palate.

**Fig. 2:**
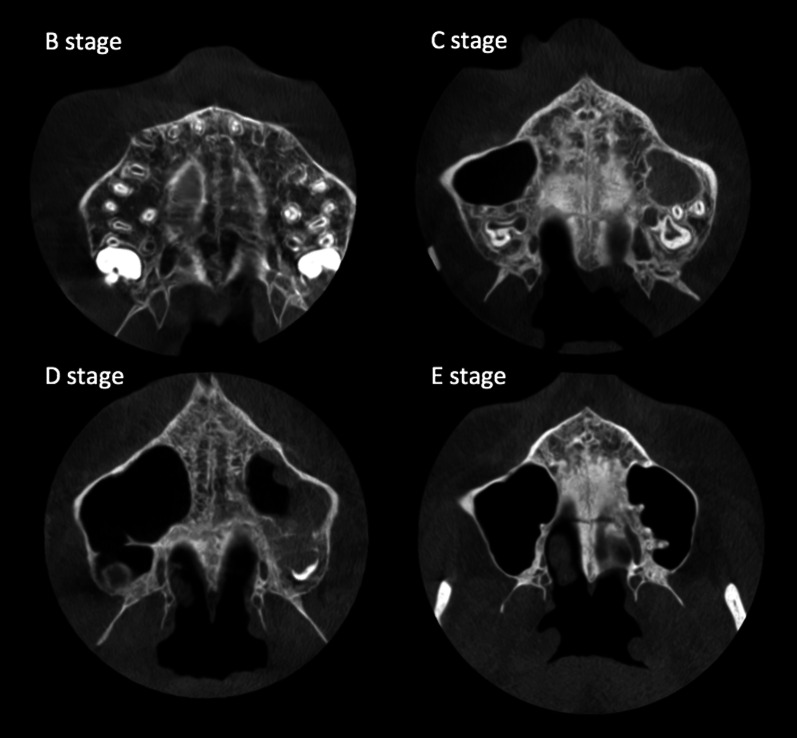
Stages B to E of maturation of the midpalatal suture (MPS) according to Angelieri et al. [12]

The classification included five stages (Fig. 2): stage A, the midpalatal suture was a straight high-density line without interdigitation; stage B, the midpalatal suture was a scalloped high-density line, with an irregular shape; stage C, the midpalatal suture appeared as two parallel, scalloped, high-density lines closed to each other; stage D, fusion of the midpalatal suture only in the palatine bone; and stage E, fusion of the midpalatal suture also in the maxillary portion of the suture and the suture was no longer visible.

The cervical vertebral maturation (CVM) stage was assessed on lateral cephalograms according to Baccetti et al. [15]. The method was based on the evaluation of C2, C3, and C4; specifically, the lower border of the body of C2, C3, and C4 (presence or absence of a concavity), and the shape of the body of C3 and C4 (trapezoid, rectangular horizontal, squared, and rectangular vertical) were analyzed.

The method includes six CVM stages (CS1-6), and it was based on the presence or absence of a concavity at the lower border of the body of C2, C3, and C4, and on the shape of the body of C3 and C4 (trapezoid, rectangular horizontal, squared, rectangular vertical).

Vertical skeletal proportions were classified according to Frankfort-mandibular plane angle (FMA) as reduced (FMA: ≤ 20 degrees), average (20 < FMA > 30 degrees) and increased (FMA: ≥ 30 degrees).

The CBCT scans were taken using Planmeca ProMax 3D (Planmeca, Helsinki, Finland) with a field of view of 100*60 mm, using 90 kV and 5.6 mA, an exposure time of 4 s, and a dose–area product of 128 mGycm^2^. The Planmeca Romexis 4.6.2 software was used for the assessment and for the reconstruction of the CBCT images. The lateral cephalograms were taken using the Proline Planmeca Dimax 4 (Planmeca, Helsinki, Finland). Cephalometric analysis was performed using Dolphin Imaging software (version 11.95; Dolphin Imaging and Management Solutions, Chatsworth, CA, USA).

One trained evaluator (MF) assessed midpalatal suture maturation, CVM stage, and FMA for all subjects with intra-examiner repeatability assessed using weighted kappa. A second evaluator (PSF) was involved in cases of uncertainty.

### Statistical analysis

Twenty images were selected randomly from the total sample and were reclassified by the same examiner one month later to assess intra-examiner agreement, and a weighted kappa coefficient was calculated to evaluate intra-examiner agreement. The normality of the data was assessed using a Shapiro–Wilk test. Continuous variables were expressed as means and standard deviations, whereas the categorical variables were presented as absolute numbers and percentages. A proportional odds logistic regression model was used to assess the association of age adjusted for sex on the midpalatal suture maturation. Association between midpalatal suture maturation and vertical skeletal dimension was assessed using regression analysis with a *P*-value of less than 0.05 being considered statistically significant. All statistical analyses were conducted with STATA® version 17 software (Stata Corporation, College Station, TX, USA).

## Results

The repeatability of the classification of the outcomes (MPS maturation stage, CVM, and FMA) was confirmed using a weighted kappa. The respective scores for MPS, CVM, and FMA were 0.885 (95% CI 0.782–0.989; *P* < 0.001), 0.856 (95% CI 0.753–0.959; *P* < 0.001), and 0.829 (95% CI 0.730–0.928; *P* < 0.001).

Out of 322 patients who had undergone both CBCT and cephalograms for orthodontic evaluation, a total of 201 patients with a mean age of 13.48 (SD 1.94) years satisfied the inclusion criteria. There were 84 male (13.38 ± 1.96 years) and 117 female (13.55 ± 1.92 years) subjects overall.

**Fig. 3:**
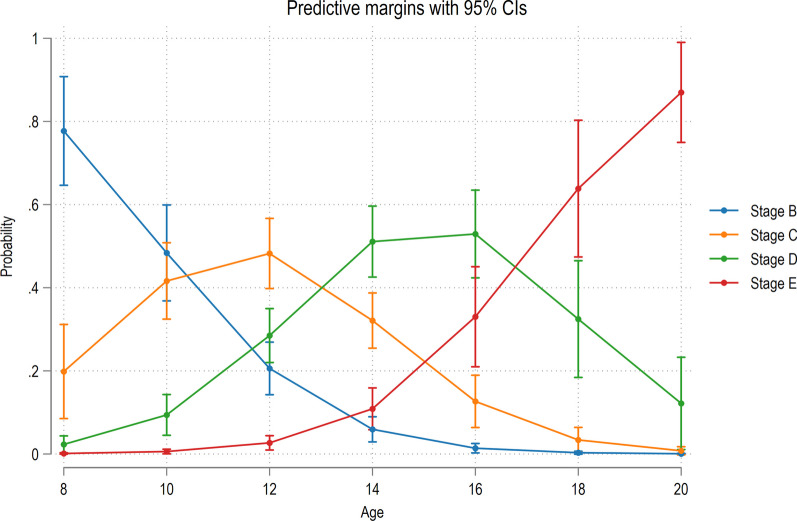
Probability of belonging to MPS stage B–E according to age

Earlier maturation of the MPS was noted in females than males. The midpalatal maturation stages D and E (suture fusion) were not reported before the age of 10 in females or before the age of 12 in males (Table 1). On the other hand, stage C was observed until 19 and 17 years, respectively, in male and female subjects. Over the age of 12 years, however, most males and females were in either stages D or E. The adjusted proportional odds logistic regression model indicated that the odds of belonging in a higher maturation stage increased with increasing age (Odds ratio: 2.14; 95% CI 1.789; 2.567; *P* < 0.001; Fig. 3).

**Table 1:** Distribution of the subjects (*n* = 201) based on MPS maturation, age, and gender

Age	MPS stage							
	B (*n* = 30)		C (*n* = 27)		D (*n* = 22)		E (*n* = 5)	
Male	*n*	%	*n*	%	*n*	%	*n*	%
8	1	3.33	0	0	0	0	0	0
9	2	6.67	0	0	0	0	0	0
10	6	20	1	3.7	0	0	0	0
11	8	26.67	3	11.11	0	0	0	0
12	6	20	5	18.53	2	9.09	0	0
13	5	16.67	7	25.93	7	31.82	1	20
14	2	6.66	8	29.63	4	18.18	1	20
15	0	0	1	3.7	4	18.18	1	20
16	0	0	1	3.7	4	18.18	1	20
17	0	0	0	0	1	4.55	1	20
19	0	0	1	3.7	0	0	0	0

Age	MPS stage							
	B (*n* = 4)		C (*n* = 45)		D (*n* = 53)		E (*n* = 15)	
Female	*n*	%	*n*	%	*n*	%	*n*	%
9	0	0	1	2.22	0	0	0	0
10	2	50	4	8.89	1	1.89	0	0
11	1	25	9	20	5	9.43	0	0
12	1	25	15	33.33	12	22.64	1	6.66
13	0	0	9	20	10	18.87	2	13.33
14	0	0	2	4.45	15	28.30	3	20
15	0	0	4	8.89	7	13.21	2	13.33
16	0	0	0	0	3	5.66	1	6.67
17	0	0	1	2.22	0	0	3	20
18	0	0	0	0	0	0	1	6.67
19	0	0	0	0	0	0	1	6.67
20	0	0	0	0	0	0	1	6.67

*MPS* midpalatal suture

In terms of skeletal maturation, all in CVM 1 stage had an MPS in either stages B and C stages. Out of 20 male patients in CVM stage 2, only one male (5%) reported an inter-digitated (stage D) suture. In both males and females, the 70% of the CVM 3 patients were in MPS stage C; however, 12 subjects (22%) were in stage D (Table 2). Approx. 64% of the CVM 4 subjects were in MPS D and E stages of MPS reflecting midpalatal suture fusion. The corresponding figure for those in CVM stage 5 was 73%, while all subjects in CVM stage 6 reported fusion of the suture (stages D and E).

**Table 2:** Distribution of the study participants based on midpalatal suture maturation stage and cervical vertebral maturation

MPS stage	CVM stage											
	CVM 1 (*n* = 12)		CVM 2 (*n* = 16)		CVM 3 (*n* = 20)		CVM 4 (*n* = 19)		CVM 5 (*n* = 16)		CVM 6 (*n* = 1)	
Male	*n*	%	*n*	%	*n*	%	*n*	%	*n*	%	*n*	%
B	12	100	14	87.5	3	15	1	5.26	0	0	0	0
C	0	0	1	6.25	14	70	8	42.11	4	25	0	0
D	0	0	1	6.25	3	15	7	36.84	11	68.75	0	0
E	0	0	0	0	0	0	3	15.79	1	6.25	1	100

MPS stage	CVM stage											
	CVM 1 (*n* = 1)		CVM 2 (*n* = 4)		CVM 3 (*n* = 30)		CVM 4 (*n* = 45)		CVM 5 (*n* = 29)		CVM 6 (*n* = 8)	
Female	*n*	%	*n*	%	*n*	%	*n*	%	*n*	%	*n*	%
B	1	100	2	50	1	3.33	0	0	0	0	0	0
C	0	0	2	50	21	70	14	31.11	8	27.59	0	0
D	0	0	0	0	8	26.67	24	53.33	16	55.17	5	62.5
E	0	0	0	0	0	0	7	15.56	5	17.24	3	37.5

*MPS* midpalatal suture, *CVM* cervical vertebral maturation

**Fig. 4:**
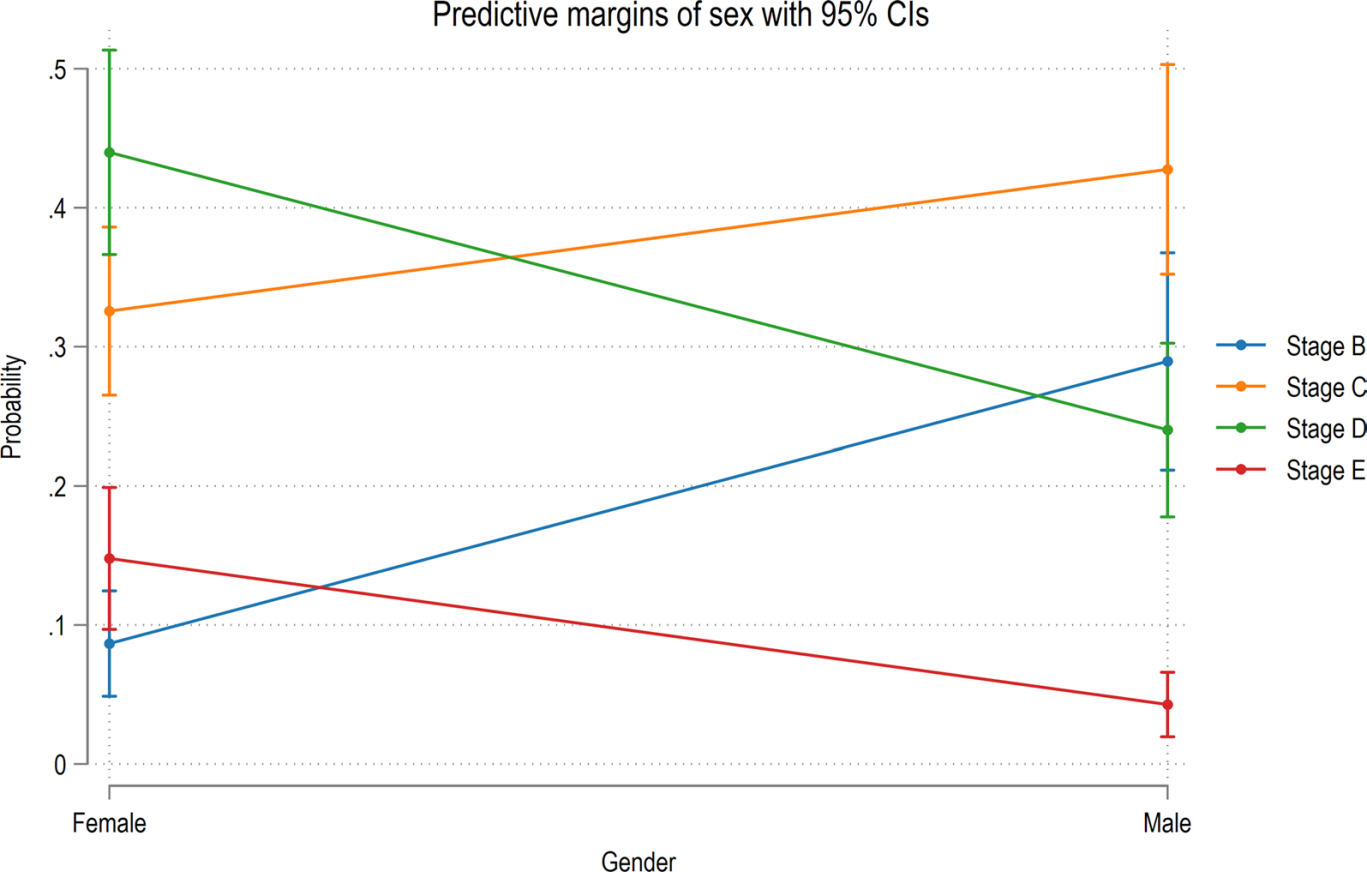
Probability of belonging to MPS stage B–E based on gender

As FMA increased, little relationship with MPS stage was observed (Table 3) with, for example, marginally more hyperdivergent males being in stages D and E (40%), while more hypodivergent females were in stages D and E (62.97%). Based on the ordinal logistic regression adjusted for gender, no association between FMA and MPS maturation was observed (odds ratio: 1.01; 95% CI 0.964; 1.051; *P* = 0.761). However, based on a further regression analysis, males had a lower probability of belonging to a higher MPS maturation stage (odds ratio: 0.24; 95% CI 0.136; 0.415; *P* < 0.001; Fig. 4).

**Table 3:** Distribution of the study participants based on midpalatal suture maturation stage and FMA

MPS stage	FMA					
	Hypodivergent (*n* = 24)		Average (*n* = 45)		Hyperdivergent (*n* = 15)	
Male	*n*	%	*n*	%	*n*	%
B	8	33.33	18	40	4	26.67
C	9	37.5	13	28.89	5	33.33
D	6	25	11	24.44	5	33.33
E	1	4.17	3	6.67	1	6.67

Average (*n* = 45)	Average (*n* = 45)					
	Hypodivergent (*n* = 27)		Average (*n* = 71)		Hyperdivergent (*n* = 19)	
Female	*n*	%	*n*	%	*n*	%
B	1	3.7	2	2.82	1	5.26
C	9	33.33	29	40.85	7	36.84
D	14	51.86	31	43.66	8	42.11
E	3	11.11	9	12.67	3	15.79

*MPS* midpalatal suture, *FMA* Frankfort-mandibular plane angle

## Discussion

The advent of alternatives to SARPE has altered the decision-making surrounding the management of transverse maxillary constriction. In particular, the use of MARPE may offer a more conservative means of sutural expansion in mature patients. Ironically, however, its use in younger patients has also grown risking excessive invasiveness. We therefore aimed to identify indicators of sutural maturity that might better inform treatment timing, particularly in the absence of CBCT imaging.

In contrast with previous studies showing a moderate to strong correlation between MPS maturation and CVM stage, we were unable to show a clear association [17, 25]. Jang et al. [25] reported a patent MPS before CVM stage 3 in females and stage 4 in males, whereas we observed the presence of stage D by CVM 2. Moreover, they stated that stage E appeared only in CVM 5 and 6, but we recorded the occurrence of stage E by CVM 4. Similarly, Angelieri et al. [13] reported that CVM 3 could be used for the diagnosis of MPS stage C. A characteristic rate of MPS maturation in hyperdivergent subjects was not observed. This finding is in contrast with a recognized delay in craniofacial growth, and later and longer mandibular growth spurt in hyperdivergent subjects [22]. Oliveira et al. [23] reported that dolichofacial patients had a higher prevalence of sutures classified in stage B and C; however, their evaluation was confined to eight patients in stages B and C, whereas we analyzed 108 patients in these two stages (27 hypodivergent, 62 average, and 17 hyperdivergent).

A significant correlation between both increasing chronological age and female gender and the probability of belonging to a higher maturation stage was observed. However, some exceptions were noted with patent sutures seen up to the ages of 17 in females and 19 in males. These results mirror previous studies with 52.3% of males and in 39.7% of females aged 16–20 years in stage C. Equally, stage C has been noted in 12–17.4% of subjects older than 30 years [12, 18, 26]. On the other hand, the results of the present study showed fusion of the MPS (stages D and E) in young patients from the age of 10 in female and 12 in male patients. Similarly, Jang et al. [25] showed that stage D did not appear before the age of 10 in female and 13 in male patients, also reaffirming that MPS maturation occurred earlier in female subjects than in male subjects.

It has been suggested based on systematic review that CBCT-based assessment of MPS may be unnecessary in patients younger than 14 years, especially in males [10]. However, our results pointed to a high prevalence of relatively early fusion with 53% of females and 22% of males having a fused midpalatal suture by 14 years. As such, it may be reasonable to recommend conventional RME in A and B stages observed before the age of 9 in females and 10 years in males. In older patients, while not mandatory, the review of CBCT images may be helpful in the evaluation of MPS maturation in order to enhance predictability and avoid unwanted side effects of midpalatal resistance including buccal tipping of the anchor teeth and fenestration of the buccal cortex [6].

The present study was cross-sectional in nature precluding the evaluation of longitudinal changes in MPS maturation or indeed the evaluation of treatment outcomes. Moreover, the subjects were recruited from a single setting which could potentially limit the generalizability of the findings. Notwithstanding these limitations, MPS maturation does not appear to be closely associated with vertical growth pattern and could not be determined on the basis of the CVM staging. As such, the use of CBCT images in female subjects older than 10 years and in males older than 12 years may be a useful diagnostic aid, although further evaluation evaluating treatment response to surgical and non-surgical approaches would be timely. Moreover, complementary prospective clinical research considering the effects of these parameters on the nature of transverse correction would be welcome. It should also be recognized that other sutures, including the zygomaticotemporal, zygomaticofrontal, and zygomaticomaxillary, might increase the mechanical resistance to sutural separation making RME less predictable with further associated research being warranted [27].

## Conclusions

Based on this cross-sectional analysis, midpalatal sutural maturation classification is associated with chronological age and is delayed in males. Neither CVM staging nor variation in vertical skeletal proportions were useful predictors of midpalatal maturation stage. As such, given the effect of midpalatal sutural resistance on treatment planning, a tailored diagnosis should be made in order to best define the optimal treatment plan to balance the degree of invasiveness with the predictability of treatment outcomes.

## Data Availability

The datasets used and/or analyzed during the current study are available from the corresponding author on reasonable request.
